# Dysregulation of the MiR-449b target TGFBI alters the TGFβ pathway to induce cisplatin resistance in nasopharyngeal carcinoma

**DOI:** 10.1038/s41389-018-0050-x

**Published:** 2018-05-22

**Authors:** Pierre-Antoine Bissey, Jacqueline H. Law, Jeff P. Bruce, Wei Shi, Aline Renoult, Melvin L. K. Chua, Kenneth W. Yip, Fei-Fei Liu

**Affiliations:** 1 Princess Margaret Cancer Centre, University Health Network, Toronto, ON Canada; 2 Department of Medical Biophysics, University of Toronto, Toronto, ON Canada; 3 LabEx DEVweCAN, Université de Lyon, F-69000 Lyon, France; 4 Department of Radiation Oncology, Princess Margaret Cancer Centre and University of Toronto, Toronto, ON Canada; 5 Division of Radiation Oncology, National Cancer Centre, Singapore, Singapore; 6 Duke-NUS Graduate School, Singapore, Singapore

## Abstract

Despite the improvement in locoregional control of nasopharyngeal carcinoma (NPC), distant metastasis (DM), and chemoresistance persist as major causes of mortality. This study identified a novel role for miR-449b, an overexpressed gene in a validated four-miRNA signature for NPC DM, leading to chemoresistance via the direct targeting of transforming growth factor beta-induced (TGFBI). In vitro shRNA-mediated downregulation of TGFBI induced phosphorylation of PTEN and AKT, increasing cisplatin resistance. Conversely, the overexpression of TGFBI sensitized the NPC cells to cisplatin. In NPC patients treated with concurrent chemoradiotherapy (CRT), the overall survival (OS) was significantly inversely correlated with miR-449b, and directly correlated with both TGFBI mRNA and protein expression, as assessed by RNA sequencing and immunohistochemistry (IHC). Mechanistically, co-immunoprecipitation demonstrated that TGFBI competes with pro-TGFβ1 for integrin receptor binding. Decreased TGFBI led to increased pro-TGFβ1 activation and TGFβ1 canonical/noncanonical pathway-induced cisplatin resistance. Thus, overexpression of miR-449b decreases TGFBI, thereby altering the balance between TGFBI and pro-TGFβ1, revealing a novel mechanism of chemoresistance in NPC.

## Introduction

Nasopharyngeal carcinoma (NPC) is an Epstein–Barr virus (EBV)-associated malignancy^1,2^ that accounts for 87,000 new cases and 51,000 deaths annually^3,4^. Patients with locally advanced disease have a 5-year overall survival (OS) rate of only about 65%^5^. While treatment with intensity-modulated radiation therapy (RT) has significantly improved locoregional control, distant metastasis (DM) remains a major clinical challenge, causing death in 20–30% of all NPC patients^6^. Furthermore, the therapeutic options for patients with locally advanced NPC remain limited, and resistance to chemoradiation remains a major clinical challenge^7^. Concurrent cisplatin or 5-fluorouracil chemotherapy in combination with RT provides only a modest improvement in OS, yet causes significant toxicity and sometimes even death^5,6,8–10^. Hence, there is an urgent need to better understand the underlying factors that drive DM and treatment resistance, which to date have remained elusive.

The transforming growth factor beta (TGFβ) pathway has been recently implicated in chemoresistance. Mechanistically, this process may occur via either an epithelial-to-mesenchymal transition (EMT)^11–13^ or the maintenance of tumor-initiating cell heterogeneity^14^. Transforming growth factor beta 1 (TGFβ1) is secreted as part of a large latent complex (LLC), which binds to, and is stored in the extracellular matrix (ECM). The LLC is composed of the mature TGFβ1 non-covalently bound to its latency-associated propeptide, forming small latent complex, which in turn is covalently attached to the large latent TGFβ1-binding protein^15^. In the ECM, the LLC may be subsequently activated when its Arg-Gly-Asp (RGD) motif binds directly to the RGD motif of the integrin receptors (e.g., αvβ3, αvβ5, and αvβ6), and the mechanical forces exerted between the ECM and integrins trigger the release of active TGFβ1^16,17^.

Among a plethora of TGFβ targets (reviewed in ref. ^18^) is transforming into growth factor beta-induced (TGFBI), a secreted protein containing four fasciclin 1 domains and an RGD motif that facilitates its interaction with integrins (i.e., αvβ3 and αvβ5)^19–24^. Similar to TGFβ1, both oncogenic and tumor suppressor functions have been reported for TGFBI^25^. Disruption of TGFBI function can lead to spontaneous tumor formation^26^ and resistance to chemotherapy^27–30^. Furthermore, TGFBI overexpression can trigger apoptosis and inhibit angiogenesis^19,31–33^. In several malignancies, however, TGFBI expression was observed to be elevated compared to normal tissues^34,35^, hence, the role of TGFBI appears to be highly dependent on the cellular context. While the TGFβ pathway has been extensively studied, the mechanisms responsible for mediating apoptosis vs. survival have not yet been completely elucidated.

Our laboratory recently completed a global microRNA (miRNA) profiling of two independent NPC cohorts, identifying and validating a four-miRNA (miR-34c, miR-140, miR-154, and miR-449b) prognostic signature for DM^36^. However, the mechanisms by which these miRNAs affect NPC biology remained to be determined. In the present study, we examined the functional role of miR-449b in NPC and identified a new miR-449b ~ TGFBI axis as a key regulator of the TGFβ pathway. Our findings demonstrate that miR-449b directly binds and represses TGFBI mRNA, altering the TGFBI-TGFβ1 balance to facilitate activation of the TGFβ pathway, thereby inducing treatment resistance in NPC.

## Results

### MiR-449b induces cisplatin resistance and targets TGFBI

**Fig. 1:**
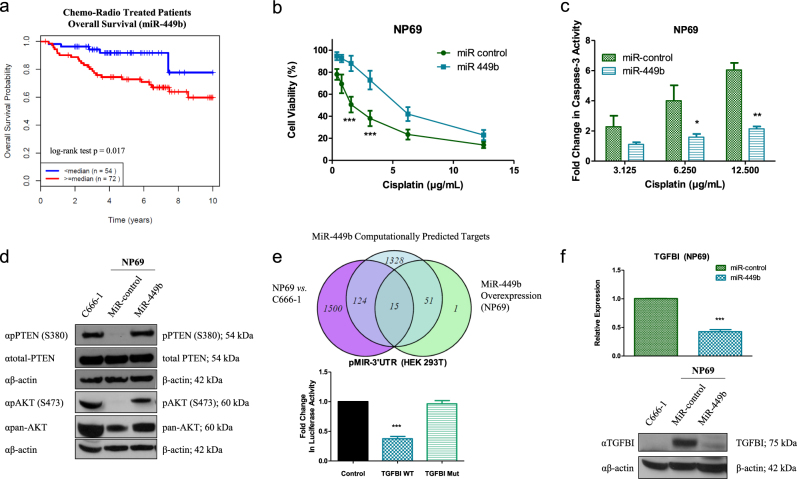
MiR-449b expression-induced cisplatin resistance and downregulated TGFBI expression. **a** Kaplan–Meier plot of OS for NPC patients dichotomized on the basis of high (≥median) vs. low (<median) miR-449b expression (*n* = 126). **b** Cell viability was assessed by ATPlite assay 72 h after cisplatin treatment in NP69-miR-449b (or control) stable cell lines. **c** Caspase-3 activity was assayed on NP69-miR-449b stable cell lines 20 h after cisplatin treatment, compared to untreated cells. **d** Western blot was performed on whole-cell lysates (WCLs) of NP69-miR-449b (or control) and C666-1 cells after 48 h of incubation in MEM. Immunoblots were performed with anti-PTEN (αtotal-PTEN), anti-phospho PTEN (S380; αpPTEN (S380)), anti-phospho AKT (S473; αpAKT (S473)), anti-pan AKT (αpan-AKT), with anti-β-actin (αβ-actin) as the loading control. **e** Top: miR-449b targets were evaluated after RNA sequencing. The Venn diagram was generated using the R package for Venn diagram software, combining miRWalk predicted targets, downregulated (4-fold) genes observed after transfection with pre-miR-449b (miR-449b), and downregulated (4-fold) genes in C666-1 vs. NP69 cells. Bottom: relative luciferase activity was assessed after transient transfection of pre-miR-449b (20 nM) for 48 h and co-transfection of pMIR-TGFBI 3′UTR wild-type (WT) (150 ng) or pMIR-TGFBI 3′UTR mutant (Mut) (150 ng) and Renilla plasmid (100 ng) for 24 h. **f** Top: relative expression of TGFBI assessed by qRT-PCR in NP69-miR-449b stable cell lines. Bottom: western blot was performed on whole-cell lysates (WCLs) of NP69-miR-449b (or control) and C666-1 cells after 48 h of incubation in MEM. Immunoblots were performed with anti-TGFBI (αTGFBI) and with anti-β-actin (αβ-actin) as a loading control. The data are expressed as the mean ± SEM of at least three independent experiments. *P < 0.05; **P < 0.01; ***P < 0.001

Given the significance of metastasis and chemoresistance in NPC^37–40^, the four-miRNA DM prognostic signature was examined in further detail for its associations with chemoresistance^36^. As shown in Fig. 1a, chemoradiotherapy (CRT)-treated NPC patients with elevated miR-449b expression experienced an inferior 5-year OS of 72.8 vs. 91.8% for low miR-449b-expressing patients (*p* = 0.017; the median follow-up time for these patients was 6.09 years, with a minimum of 0.96 years and a maximum of 13.5 years). This observation motivated a further examination of the role of miR-449b in vitro. C666-1, an EBV-positive NPC cell line, exhibited higher endogenous miR-449b, compared to both NP69 and NP460, two immortalized normal nasopharyngeal cell lines (Supplementary Figure S1a). Since the most common chemotherapeutic agent used in NPC is cisplatin^7,41^, it was hypothesized that miR-449b might be responsible for drug resistance. Indeed, C666-1 cells were more resistant to cisplatin, compared to both NP69 and NP460, as assessed by cell viability (Supplementary Figure S1b) and caspase-3 activity (Supplementary Figure S1c). NP69 cells stably transfected with miR-449b (NP69-miR-449b) recapitulated the increased cell viability (Fig. 1b) and lower caspase-3 activity (Fig. 1c) in response to cisplatin, compared to the control cells stably transfected with scrambled miRNA (NP69-miR-control).

In order to identify the signaling pathway that might contribute to cisplatin resistance observed in NP69-miR-449b and C666-1 cell lines, key proteins of well-established molecular pathways involved in NPC were investigated including the ERK1/2, SAPK/JNK, and PTEN/AKT pathways^42^. As can be seen in Supplementary Figure S1d and e, C666-1 cells exhibited higher ERK1/2 and SAPK/JNK activity, compared to normal NP69 and NP460 cells, but no significant changes were observed in NP69-miR-449b cells. The expression of p38 was also assessed, but phosphorylation was not detected by western blot. Greater levels of PTEN tail phosphorylation (Ser380) (Fig. 1d, Supplementary Figure S1f, Supplementary Figure S5a), which has been reported to inactivate PTEN by inducing a closed conformation^43,44^, were observed in both C666-1 and NP69-miR-449b cells. This closed conformation was indeed accompanied by higher levels of phospho-AKT (Ser473) (Fig. 1d, and Supplementary Figure S1f, Supplementary Figure S5a), suggesting one mechanism by which chemoresistance is induced in NPC cells. Indeed, the treatment of cisplatin-resistant NP69-miR-449b cells with MK-2206, an AKT inhibitor, restored the sensitivity to cisplatin (to a level similar of their control; Supplementary Figure S1g).

In order to identify candidate mRNA targets of miR-449b, NP69 cells were transfected with pre-miR-449b, followed by RNA sequencing using the Illumina HiSeq 2000. At the intersection of: (1) genes downregulated in the transfected cells; (2) computationally predicted targets; and (3) genes downregulated in C666-1 (high endogenous miR-449b) vs. NP69 (low endogenous miR-449b) cells were 15 genes (Fig. 1e, top, Supplementary Figure S2a). Amongst these genes was TGFBI, which when upregulated, has been reported to decrease AKT activity and induce chemosensitivity^27,28,30,45^. Based on these observations, we decided to focus our study on TGFBI and its potential role in NPC chemoresistance. First, direct targeting of TGFBI by miR-449b was confirmed using a luciferase reporter assay (Fig. 1e, bottom). These findings were further corroborated as NP69-miR-449b cells expressed lower levels of TGFBI, as observed by both quantitative real-time PCR (qRT-PCR) (Fig. 1f, top; Supplementary Figure S2b; transient transfection), as well as western blotting for cytosolic (Fig. 1f, bottom, Supplementary Figure S5a) and secreted (Supplementary Figure S2c) protein expression, when compared to NP69-miR-control. Inhibition of miR-449b led to an increase of TGFBI expression at both the RNA level (Supplementary Figure S2d, top, stable cells; bottom, transient transfection), and protein level: cytosolic (Supplementary Figure S2e, left) and secreted (Supplementary Figure S2e, right). Moreover, NP69 cells stably expressing a miR-449b inhibitor (NP69-anti-miR-449b) had increased the sensitivity to cisplatin (Supplementary Figure S2f), confirming that miR-449b, and potentially TGFBI, has a role in cisplatin resistance in NPC cells.

### TGFBI increases cisplatin sensitivity in NP69 cells

**Fig. 2:**
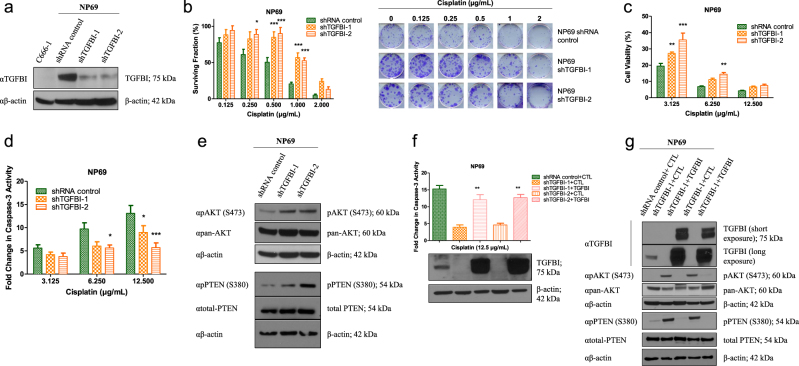
TGFBI regulates cisplatin sensitivity of normal nasopharyngeal cells. Experiments were performed using stable cell lines generated by lentivirus infection. **a** Western blot of NP69-shTGBI (−1, −2) C666-1 (infected with shRNA control) whole-cell lysates (WCLs) after 48 h of incubation in MEM media. Immunoblots were performed with anti-TGFBI (αTGFBI), with anti-β-actin (αβ-actin) as the loading control. **b** Clonogenic assay of NP69-shTGFBI (−1, −2) cells treated with cisplatin for 10 days. **c** Cell viability using ATPlite assay on NP69-shTGBI (−1, −2) cells treated with cisplatin for 72 h. **d** Caspase-3 activity assay on NP69-shTGFBI (−1, −2) cells after 20 h of cisplatin treatment. **e** Western blot of NP69-shTGFBI (−1, −2) WCLs after 48 h of incubation in MEM. Immunoblots were performed with anti-PTEN (αtotal-PTEN), anti-phospho PTEN (S380; αpPTEN (S380)), anti-phospho AKT (S473; αpAKT (S473)), anti-pan AKT (αpan-AKT), with anti-β-actin (αβ-actin) as the loading control. **f** Caspase-3 activity assay on NP69-shTGFBI (−1, −2) cells 48 h after transfection with empty vector (CTL) or TGFBI expression vector (Myc-tagged; TGFBI-Myc). Media was replaced with MEM 6 h after transfection, and then treated for 20 h with cisplatin. Anti-Myc (αMyc-TGFBI) and anti-β-actin (αβ-actin) immunoblots are shown as controls for specificity and loading. **g** Western blot of NP69-shTGFBI WCLs transfected with TGFBI 48 h after transfection and MEM incubation. Immunoblots were performed with anti-TGFBI (αTGFBI), anti-PTEN (αtotal-PTEN), anti-phospho PTEN (S380; αpPTEN (S380)), anti-phospho AKT (S473; αpAKT (S473)), anti-pan AKT (αpan-AKT), and anti-β-actin (αβ-actin) as the loading control. The data are expressed as the mean ± SEM of at least three independent experiments. *P < 0.05; **P < 0.01; ***P < 0.001

In order to further evaluate the role of TGFBI in cisplatin sensitivity, NP69 cells were stably infected with lentiviral vectors containing two different shRNAs targeting TGFBI (NP69-shTGFBI-1 and NP69-shTGFBI-2) (Fig. 2a, Supplementary Figures S2g and h, Supplementary Figure S5b). Both cell lines exhibited increased clonogenicity and cell viability after cisplatin treatment, when compared to the controls (Fig. 2b, c). Furthermore, both cell lines exhibited decreased caspase-3 activity following cisplatin treatment (Fig. 2d). TGFBI repression induced phosphorylation (Ser380) and inhibition of PTEN, as well as phosphorylation (Ser473) and activation of AKT (Fig. 2e, Supplementary Figure S5b). These data corroborate the results observed with miR-449b overexpression (Fig. 1), suggesting a pivotal role of TGFBI inhibition in miR-449b-mediated cisplatin resistance.

Re-expression of TGFBI in NP69-shTGFBI cells restored their sensitivity to cisplatin to a similar level as NP69 control cells (Fig. 2f). In addition, TGFBI re-expression in NP69-shTGFBI cells abolished PTEN phosphorylation, leading to PTEN activation and reduced downstream AKT phosphorylation (Fig. 2g, Supplementary Figure S5c). Taken together, these data indicate that TGFBI modulates their sensitivity to cisplatin via regulation of the PTEN-AKT pathway.

### TGFBI triggers apoptosis and restores cisplatin sensitivity in NPC

**Fig. 3:**
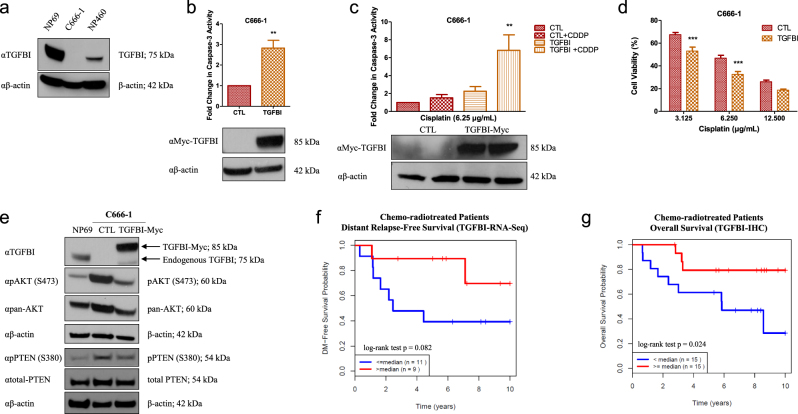
TGFBI triggers apoptosis and restores cisplatin sensitivity in NPC. **a** Western blot was performed on the whole-cell lysates (WCLs) of C666-1, NP69, and NP460 cells. Anti-TGFBI (αTGFBI) was used to assess endogenous TGFBI expression and anti-β-actin (αβ-actin) as the loading control. **b** Caspase-3 activity assay on C666-1 cells transfected with either empty vector (CTL) or TGFBI expression vector (Myc-tagged; TGFBI-Myc) for 48 h. Media was replaced with MEM 6 h after transfection. **c** Caspase-3 activity after incubation with cisplatin (CDDP) for an additional 20 h. Anti-Myc (αMyc-TGFBI) and anti-β-actin (αβ-actin) immunoblots are shown as controls for specificity and loading. **d** Cell viability, using ATPlite, of C666-1 cells transfected with empty vector (CTL) or TGFBI-expression vector (TGFBI-Myc), incubated for 24 h, re-seeded in 96-well plates, and treated with cisplatin for 72 h in MEM. **e** Western blot of NP69, or C666-1 WCLs 48 h after transfection with empty vector (CTL) or TGFBI-expression vector (Myc-tagged; TGFBI-Myc). Media was replaced with MEM 6 h after transfection. Immunoblots were performed using anti-TGFBI (αTGFBI), anti-PTEN (αtotal-PTEN), anti-phospho PTEN (S380; αpPTEN (S380)), anti-phospho AKT (S473; αpAKT (S473)), anti-pan AKT (αpan-AKT), and anti-β-actin (αβ-actin) as the loading control. The data are expressed as the mean ± SEM of at least three independent experiments. *P < 0.05; **P < 0.01; ***P < 0.001. **f** Kaplan–Meier plot of DRFS for NPC patients dichotomized on the basis of high (>median) vs. low (≤median) TGFBI mRNA expression level (*n* = 20). **g** IHC quantification of non-relapsed vs. relapsed NPC patient samples using an anti-TGFBI polyclonal antibody. The Kaplan–Meier curve was generated based on high (≥median) vs. low (<median) TGFBI expressed (cytoplasmic and membrane staining of tumor cells) (*n* = 30)

Since TGFBI is not endogenously expressed in C666-1 cells (Fig. 3a, and Supplementary Figures S3a and b), this model was utilized to assess the consequences of TGFBI overexpression. As anticipated, TGFBI overexpression in C666-1 cells triggered caspase-3 activation (Fig. 3b), corroborating the previously published results demonstrating the pro-apoptotic properties of TGFBI^32,33^. Concordantly, TGFBI overexpression sensitized C666-1 cells to cisplatin (Fig. 3c, d; C666-1 cells are not suitable for clonogenic assays as they do not form colonies), activated PTEN (through reduced phospho-PTEN Ser380), and reduced AKT phosphorylation (low phospho-AKT-S473; Fig. 3e, Supplementary Figure S5d).

In order to investigate the clinical significance of TGFBI in NPC, RNA sequencing was performed on the diagnostic biopsy samples from NPC patients treated with CRT (*n* = 20). As anticipated, patients with elevated TGFBI experienced a superior 5-year distant relapse-free survival (DRFS) of 90 vs. 40% for patients with low TGFBI expression (*p* = 0.082; Fig. 3f). This observation was further confirmed using immunohistochemistry (IHC; *n* = 30), wherein patients with greater than median TGFBI immunostaining (cytoplasmic or membrane) had a higher 5-year OS of 80 vs. 60% for patients with low TGFBI immunoexpression (*p* = 0.024; Fig. 3g, Supplementary Figure S3c), corroborating the clinical significance of TGFBI in mediating NPC survival.

### TGFBI downregulation triggers AKT and EMT-like pathways via TGFβ

**Fig. 4:**
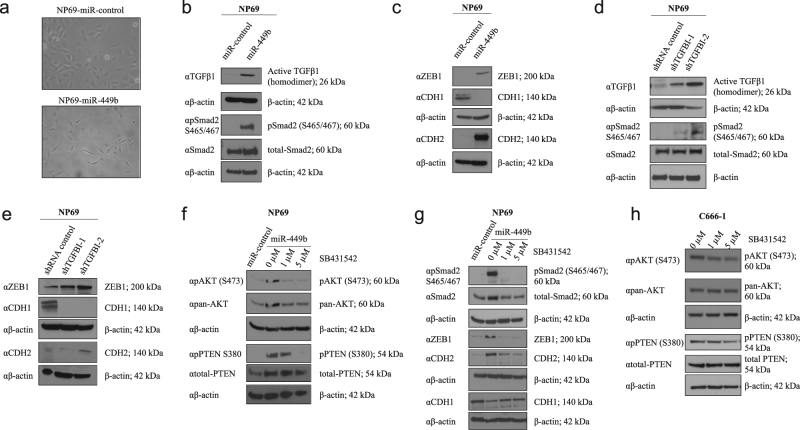
TGFBI downregulation triggers AKT and EMT-like pathways via TGFβ. **a** Representative photographs of NP69 stably transfected cells. NP69-miR-control cells exhibited epithelial morphology, while NP69-miR-449b cells had an elongated shape. Both photographs were taken with the Leica DMIRB microscope using the NPLAN L ×20/0.40 CORR PH1 lens, and have been cropped. Original uncropped photographs are shown in Fig S3d. **b**–**e** Western blots were performed on NP69-miR-449b stable (b and c) and NP69-shTGFBI stable (**d**, **e**) cells incubated in MEM for 48 h. **f**, **g** NP69-miR-449b stable cells incubated in MEM were treated with SB431542, a TGFBR1 inhibitor, at 1 µM or 5 µM for 72 h. **h** C666-1 cells were incubated for 72 h with SB431542, a TGFBR1 inhibitor, at 1 or 5 µM in MEM. C666-1 cells do not express ZEB1 and CDH2. **b**, **c**, **d**, **e**, **g** Anti-TGFB1 (αTGFβ1), anti-phospho SMAD2 (S465/467; αpSMAD (S465/467)), and anti-SMAD2 (αSMAD2) antibodies was used to assess the expression of active TGFβ1 and canonical TGFβ pathway activation, while anti-ZEB1 (αZEB1), anti-CDH1 (αCDH1), and anti-CDH2 (αCDH2) antibodies were used as indicators of EMT. **f**, **h** Anti-PTEN (αtotal-PTEN), anti-phospho PTEN (S380; αpPTEN (S380)), anti-phospho AKT (S473; αpAKT (S473)), and anti-pan AKT (αpan-AKT) antibodies were used. Anti-β-actin (αβ-actin) antibody was used as the loading control. The data are expressed as the mean ± SEM of at least three independent experiments

A change in cellular morphology was observed during the generation of miR-449b stably transfected cells, wherein NP69-miR-449b cells exhibited a more elongated and mesenchymal-like shape, compared to NP69-miR-control cells (Fig. 4a, Supplementary Figure S3d). Thus, the role of EMT in cisplatin resistance was investigated in these models. Because TGFβ1 is a known inducer of EMT and is associated with TGFBI expression^21,32^, its expression was first evaluated in these cells. Higher intracellular levels of active TGFβ1 associated with Smad2 phosphorylation were observed in NP69-miR-449b cells, in comparison to the control cells (Fig. 4b, Supplementary Figure S5e). This increase in active intracellular TGFβ1 was accompanied by increased expression of the transcriptional repressor ZEB1 and decreased expression of the epithelial marker E-cadherin (CDH1) at both the protein (Fig. 4c, Supplementary Figure S5e) and mRNA levels (Supplementary Figure S3e). Moreover, two other mesenchymal markers vimentin (VIM; mRNA level; Supplementary Figure S3e) and N-cadherin (CDH2; protein and mRNA levels; Fig. 4c, Supplementary Figure S3e, Supplementary Figure S5e) were also upregulated.

Both NP69-shTGFBI cell lines exhibited a similar molecular phenotype: (1) greater levels of active intracellular TGFβ1 associated with phospho-Smad2 expression (Fig. 4d, Supplementary Figure S5f); (2) higher expression of the mesenchymal markers ZEB1, CDH2 (protein and mRNA levels; Fig. 4e, Supplementary Figure S3f, Supplementary Figure S5f), and VIM (mRNA level; Supplementary Figure S3f); and (3) lower CDH1 expression (protein and mRNA levels; Fig. 4e, Supplementary Figure S3f, Supplementary Figure S5f). Although, these changes were not accompanied by morphological alterations, they suggest that TGFBI downregulation promotes or contributes to an EMT-like phenotype (the transcriptional expression of Snail and Slug, common promoters of EMT induced by TGFβ1, were unchanged in both models; data not shown). Transient overexpression of TGFBI in C666-1 cells diminished the intracellular expression of active TGFβ1 (Supplementary Figure S3g; analysis of EMT-like phenotype was not possible as C666-1 cells do not express ZEB1 and CDH2).

In order to evaluate the significance of active TGFβ1 expression in our model, NP69-miR-449b cells were treated with the TGFBR1 inhibitor SB431542 (1 and 5 µM). This treatment resulted in the reduction of phosphorylated AKT (S473), phosphorylated PTEN (S380) (Fig. 4f, Supplementary Figure S5g), phosphorylated Smad2, ZEB1, and CDH2, whereas CDH1 was slightly upregulated (Fig. 4g, Supplementary Figure S5h), which confirmed previous studies suggesting that the inhibition of PTEN by TGFβ1 was the cause of TGFβ-dependent AKT activation^46,47^. In addition, C666-1 cells treated with SB431542 reduced AKT phosphorylation (S473) and PTEN phosphorylation (Fig. 4h, Supplementary Figure S5i), a phenotype similar to that observed after TGFBI overexpression (Figs. 2g and 3e). This further corroborated a significant role of TGFβ1 in AKT and PTEN phosphorylation, leading to chemoresistance in C666-1 cells. However, treatment of NP69-miR-449b cells with MK-2206 (the AKT inhibitor) did not affect the expression of ZEB1, CDH1, or CDH2 (Supplementary Figure S3h). Taken together, these data indicate that when TGFBI is downregulated, TGFβ1 causes: (1) PTEN inhibition and AKT activation; and (2) increased ZEB1 and CDH2 expression, along with decreased CDH1. In summary, the absence of TGFBI alone, or its absence mediated by miR-449b, increased chemoresistance partially through the regulation of an EMT-like TGFβ1-dependent mechanism and through PTEN/AKT regulation in these cellular models of NPC.

### TGFBI regulates TGFβ signaling by binding ITGB3/5

The binding of pro-TGFβ1 to integrins has been shown to trigger the release of active TGFβ1^17,48,49^, enabling it to bind to its receptors, inducing their phosphorylation, and triggering the canonical Smad2/3 and noncanonical signaling pathways^18,50^. Because TGFBI binds to integrin αvβ3/5^19,22–24^, we hypothesized that TGFBI competes with pro-TGFβ1 for integrin αvβ3/5 binding, leading to a decrease in TGFβ signaling when TGFBI is overexpressed, and an increase in TGFβ signaling when TGFBI is downregulated.

**Fig. 5:**
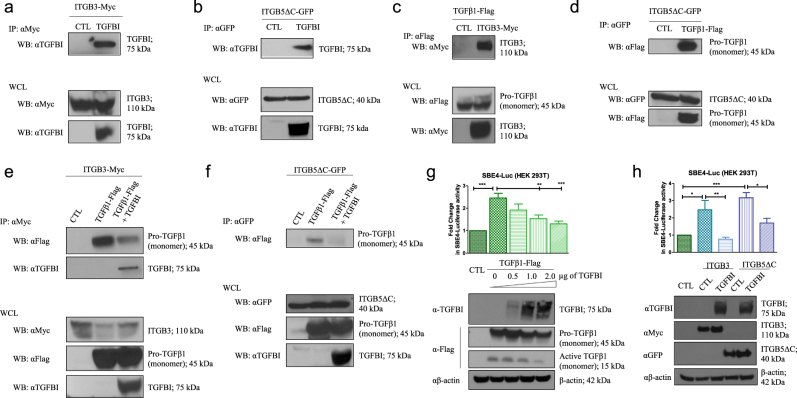
TGFBI regulates signaling pathways by binding ITGB3 and ITGB5. **a** ITGB3 immunoprecipitation (IP: αMyc) was performed on HEK293T cells expressing ITGB3 in the absence or the presence of TGFBI (via transfection). Interaction between ITGB3 and TGFBI was detected using anti-TGFBI (αTGFBI) antibody. **b** ITGB5 immunoprecipitation (IP: αGFP) was performed on HEK293T cells expressing ITGB5ΔC with or without presence of TGFBI (via transfection). Interaction between ITGB5 and TGFBI was detected using anti-TGFBI (αTGFBI) antibody. **c** Immunoprecipitation of pro-TGFβ1 (IP: αFlag) was performed on HEK293T cells expressing pro-TGFβ1 alone or with ITGB3. Interaction between ITGB3 and pro-TGFβ1 was identified using anti-Myc (αMyc) antibody. **d** Immunoprecipitation of ITGB5 (IP: αGFP) was performed on HEK293T cells expressing ITGB5ΔC alone or with pro-TGFβ1. Interaction between ITGB5 and pro-TGFβ1 was revealed using anti-Flag (αFlag) antibody. **e** Co-immunoprecipitations were performed on HEK293T cells expressing ITGB3 alone or with pro-TGFβ1 in the absence (TGFβ1-Flag) or presence of TGFBI (TGFβ1-Flag + TGFBI). These data show competition between TGFBI and pro-TGFβ1 for ITGB3 binding. **f** Co-immunoprecipitations were performed on HEK293T expressing ITGB5ΔC alone or with pro-TGFβ1 in the absence (TGFβ1-Flag) or presence of TGFBI (TGFβ1-Flag + TGFBI). These data show competition between TGFBI and pro-TGFβ1 for ITGB5 binding. For **a–f** Western blot of the whole-cell lysates (WCLs) before pull-down is shown as a control for specificity and expression. **g** Top: relative luciferase activity was assessed after transient transfection of pro-TGFβ1-Flag and TGFBI-Myc, as indicated, and the co-transfection of pSBE4-luciferase vector (150 ng) and Renilla plasmid (100 ng). Bottom: western blot of HEK293T cells transfected with pro-TGFβ1-Flag encoding plasmid (1 µg) and TGFBI-Myc encoding plasmid, as indicated. Anti-Myc (αMyc) antibody was used to assess the expression of TGFBI, and anti-Flag (αFlag) antibody was used to assess the expression of pro-TGFβ1 and active TGFβ1 forms. The expression of pro-TGFβ1 indicates similar transfection efficiency across the conditions. Anti-β-actin (αβ-actin) antibody was used as the loading control. **h** Top: relative luciferase activity was assessed after transient transfection of ITGB3-Myc (1 µg) or ITGB5ΔC-GFP (1 µg) and TGFBI (2 µg), and the co-transfection of pSBE4-luciferase vector (150 ng) and Renilla plasmid (100 ng). Bottom: western blot control of the plasmids expression: anti-TGFBI (αTGFBI) antibody was used to assess the expression of TGFBI, anti-Myc (αMyc), and anti-GFP (αGFP) for ITGB3 and ITGB5, respectively. Anti-β-actin (αβ-actin) antibody was used as the loading control. The data are expressed as the mean ± SEM of at least three independent experiments. *P < 0.05; **P < 0.01; ***P < 0.001

To validate this relationship, the interaction between TGFBI and integrin αvβ3/5 was first confirmed with pull-downs of β3- (ITGB3) and β5-integrin (ITGB5) in 293T cells expressing ITGB3 and ITGB5ΔC (β5-integrin without its cytoplasmic tail) (via transfection), respectively (Fig. 5a, b). Secondly, co-immunoprecipitation was used to demonstrate that pro-TGFβ1 binds to ITGB3 and ITGB5ΔC (Fig. 5c, d). Finally, overexpression of TGFBI led to a decrease in the interaction between pro-TGFβ1 and ITGB3/5, confirming that TGFBI does indeed compete with pro-TGFβ1 for binding to ITGB3/5 (Fig. 5e, f). To our surprise, overexpression of pro-TGFβ1 did not disrupt the interaction between TGFBI and ITGB3/5 in our model (Supplementary Figure S4a and b), suggesting that TGFBI could have a greater affinity to ITGB3/5 than pro-TGFβ1.

Ligand-receptor complex internalization has been shown to be an important component of both TGFβ pathway induction and the molecular turnover of active TGFβ1 and its receptors^51,52^. Thus, to determine the effects of competition between TGFBI and pro-TGFβ1, we assessed Smad3/4 activity using the pSBE4-luc vector (luciferase vector reporter containing four tandem copies of Smad-binding element) and the transcription of PAI-1 (a well-characterized target of TGFβ1), after co-transfection of pro-TGFβ1 with increasing amounts of TGFBI. In the absence of TGFBI, the pSBE4-luc vector was activated (Fig. 5g; top) and PAI-1 was transcribed (Supplementary Figure S4c), which corroborated the high levels of active TGFβ1 observed by western blot (Fig. 5g; bottom). In addition, increasing levels of TGFBI led to a decrease in luciferase activity of the pSBE4-luc vector (Fig. 5g; top), with a corresponding reduction in the level of intracellular active TGFβ1 (Fig. 5g; bottom) and PAI-1 transcription (Supplementary Figure S4c). In our controls, we observed a reduction in luciferase activity of the pSBE4-luc vector after SB431542 treatment (Supplementary Figure S4d; left), with no induction of luciferase activity of the pSBE-luc vector (vector reporter containing one copy of Smad-binding element) after transfection of pro-TGFβ1 (Supplementary Figure S3d; right). Interestingly, this finding corroborated the observations in Fig. 4b, d, whereby TGFBI repression was accompanied by high intracellular levels of active TGFβ1 and phospho-Smad2 expression. Additionally, we investigated whether the overexpression of TGFBI could affect the endogenous TGFβ1 pathway activation induced by exogenous ITGB3/5 expression. As seen in Fig. 5h, overexpression of either ITGB3 or ITGB5ΔC triggered the induction of the pSBE4-luc vector (but not pSBE-luc (Supplementary Figure S4e; right)), whereas TGFBI expression caused its inhibition. This corroborated our hypothesis of TGFβ1 pathway inhibition via the binding of TGFBI to ITGB3/5. As a control, SB431542 exposure inhibited the TGFβ1 pathway activity induced by ITGB3/5 (Supplementary Figure S4e; left). Taken together, these data demonstrate an important role of TGFBI in controlling TGFβ1 pathway activity via its binding with ITGB3/5.

In summary, TGFBI-ITGB3/5 binding controls AKT phosphorylation via two mechanisms: (1) activation of PTEN via its dephosphorylation; and (2) competition with pro-TGFβ1 in the ECM. Furthermore, the phenotypic changes observed in this study suggest that the TGFBI-TGFβ1 balance is critical for TGFβ pathway activation, which is also concordant with studies reporting the importance of the TGFβ pathway in EMT and chemoresistance^11–14,53^.

## Discussion

**Fig. 6:**
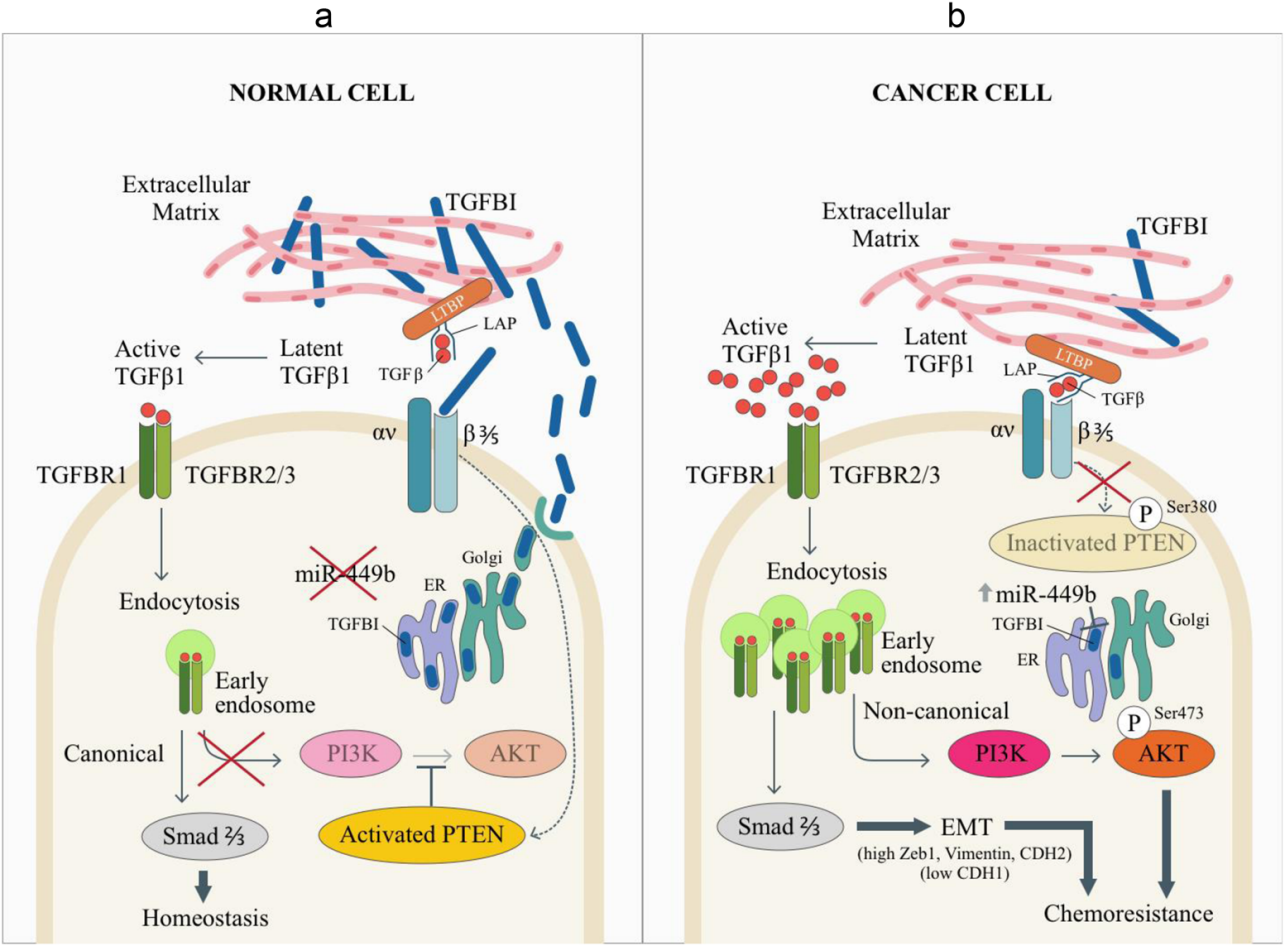
Proposed model for the miR-449b-TGFBI/TGFβ1 pathway. **a** In normal cells, the secretion of TGFBI into the ECM leads to competition between TGFBI and latent TGFβ1 (ECM-sequestered) for the binding of αvβ3/5 integrins. TGFBI-αvβ3/5 binding activates the tumor suppressor PTEN (dotted arrow; uncharacterized mechanism), suppressing AKT activation. This binding also prevents excess conversion of latent TGFβ1 into active TGFβ1, thereby modulating endocytosis of active TGFβ1 and its receptors, and maintaining homeostasis. **b** In NPC, miR-449b is overexpressed and downregulates TGFBI expression. Consequently, with less TGFBI in the ECM, this facilitates PTEN inhibition, as well as increased binding of latent TGFβ1 to αvβ3/5 integrins (via its LAP domain), promoting the release of active TGFβ1. As more active TGFβ1 binds to its receptors, it becomes internalized, resulting in increased activation of both canonical and noncanonical TGFB signaling, as demonstrated by the EMT-like phenotype acquired by the cells, as well as activation of AKT; two features known to be involved in chemoresistance^53^

This study has demonstrated the importance of the balance between TGFBI and TGFβ1 in mediating chemotherapy resistance via the TGFβ pathway. We showed that overexpression of miR-449b resulted in TGFBI inhibition and reduction of TGFBI secretion, allowing for increased integrin-mediated active TGFβ1 release, with subsequent TGFβ1 canonical/noncanonical pathway activation (Fig. 6). Previous studies have reported elevated TGFβ1 in NPC patient sera^54^, although the source of the secretion remained unclear. Interestingly, two EBV proteins, latent membrane protein 1 and EBV-encoded nuclear antigen (EBNA1), have been reported to abolish canonical TGFβ-Smad signaling^55–57^, while inducing TGFβ1 expression in vitro^56,58^. A synergistic effect between EBV infection and TGFβ1 treatment on EMT has also been reported in lung epithelial A549 cells^59^, and several EMT-related genes are elevated in NPC^60–62^.

The interplay between TGFBI and TGFβ1 that we have identified has additional importance from a regulatory perspective. Treatment with TGFβ1 may induce the expression of TGFBI^21^, suggesting a feedback mechanism by which TGFβ1 might induce apoptosis and prevent further activation. This hypothesis is supported by the fact that pro-TGFβ1 did not seem to displace TGFBI from binding to ITGB3/5, but this will require further investigation. In addition, EBNA1 has been described as a repressor of canonical TGFβ signaling, promoting Smad2 turnover and TGFBI downregulation^57^. In our current study, TGFBI expression is suppressed by miR-449b, which in turn, allows the cell to ensure TGFβ1-mediated survival.

Two pathways responsible for cisplatin resistance resulting from high miR-449b and low TGFBI-expression levels were identified in our cell line models: (1) inactivated PTEN with increased phosphorylated AKT; and (2) increased ZEB1 and N-cadherin with decreased E-cadherin (which commonly induces an EMT-like phenotype)^53^. Although, such phenotypic changes have already been described for both TGFBI and TGFβ1 independently^19,27,45,50^, the mechanisms linking TGFBI to TGFβ1 have not been elucidated until now. Here we demonstrated the competition between TGFBI and pro-TGFβ1 for ITGB3/5 binding, possibly via their RGD domains. Thus, the repression of TGFBI caused by elevated miR-449b leads to an imbalance in integrin binding. The paucity of TGFBI allows for increased pro-TGFβ1 activation via integrin binding. Thus, more active TGFβ1 will be released, leading to increased activity of the canonical and noncanonical TGFβ pathways. Further investigation is needed to determine the specific role of ITGB3/5 in TGFBI-dependent apoptosis in NPC. One hypothesis could be that ITGB3/5 serves as an anchor for the cleavage of TGFBI, releasing its C-terminal domain, leading to apoptosis.

This study also identified miR-449b and TGFBI as promising biomarkers for cisplatin response in NPC. The results are consistent with a previous study reporting that TGFBI is a favorable prognostic factor for survival in patients with squamous cell lung cancer treated with adjuvant platinum-based chemotherapy^29^. Future evaluations may also include assaying patient sera levels of TGFBI, given that it is a secreted protein; hence could potentially serve as a circulating biomarker of chemosensitivity. Finally, the importance of the TGFBI-TGFβ1 balance that we report here for NPC warrants further exploration in other human malignancies.

## Materials and methods

### Cell culture, transfection, and infection

The human normal nasopharyngeal cell lines, NP69 (immortalized by SV40) and NP460 (immortalized by hTert), were a kind gift from George S.W. Tsao (University of Hong Kong). NP69 cells were maintained in keratinocyte-free media supplemented with EGF and pituitary serum (Invitrogen, Carlsbad, CA, USA); NP460 cells were grown in keratinocyte-free media mixed with Opti-MEM (Invitrogen) at a 1:1 ratio. C666-1 (EBV-positive NPC) cells were cultured in RPMI media supplemented with 10% FBS. HEK293T, used for functional analysis and virus production, were cultured in DMEM with 10% FBS. Each new frozen batch was tested for mycoplasma (MycoAlert Mycoplasma Detection Kit, Lonza, Basel, Switzerland); after 10–11 passages, cells were discarded and a new batch was used. NP69 and C666-1 cell lines were authenticated using polymorphic short tandem repeat method and RNA sequencing. Transient transfections for co-immunoprecipitation and virus production were performed using calcium phosphate. JetPRIME (Polyplus-transfection, Graffenstaden, France) was used to transfect C666-1 for cell death assays and functional analysis in HEK293T. pCMV6-TGFBI-Myc (Origene, Rockville, MD, USA), pCMV6-XL5-TGFBI non-tagged (Origene), pcDNA3.1-beta-3 (Addgene plasmid #27289; from Timothy Springer, Addgene, Cambridge, MA, USA), pCX-EGFP beta5ΔC (Addgene plasmid #14996; from Raymond Birge), pCMV3-TGFB1-Flag (Sino Biological Inc., Beijing, China), pSBE4-Luc (Addgene plasmid #16495; from Bert Vogelstein^63^), pSBE-Luc (Addgene plasmid #16527; from Bert Vogelstein^63^), and pcDNA.3.1-Flag were used. NP69 and NP460 cells were transfected with pre-miR-449b (Ambion, Austin, TX, USA; 20 nM), while pre-miR negative control (Ambion; 20 nM) was transfected using JetPRIME.

Stable cell lines were generated using lentiviral infection. pLV-miRNA-449b (Biosettia, San Diego, CA, USA), pLKO.1 shRNA-TGFBI-1 (TRCN0000062174) and shRNA-TGFBI-2 (TRCN0000062176) (Sigma, St. Louis, MO, USA), and their respective control vectors were used. The plasmids were transfected into HEK293T with psPAX2 and pCMV-VSV-G (Addgene) for virus encapsulation. After an overnight incubation, media was changed, and then at 48 and 72 h after transfection, the media was collected and changed, centrifuged, and filtered (0.45 µm filter). NP69 cells were then incubated for 24 h with each media condition; 48 h later, cell selection was performed using puromycin or blasticidin.

SB431542 (#S1067, SelleckChem, Houston, TX, USA) is a selective inhibitor of ALK5, also known as TGFβ receptor I. MK-2206 (#S1078, SelleckChem) is a selective inhibitor of AKT1/2/3.

### Western blot and co-immunoprecipitation

For both western blot and co-immunoprecipitation, proteins were extracted using immunoprecipitation buffer (50 mM Hepes pH 7.6, 150 mM NaCl, 5 mM EDTA, 1–2% Nonidet P-40) in the presence of protease inhibitor cocktail (Roche, Basel, Switzerland), and then separated using a Bolt 4–20% Gel (Life Technologies, Carlsbad, CA, USA). For immunoprecipitation, the lysates were incubated overnight at 4 °C either with anti-GFP (Santa Cruz Biotechnology, Dallas, TX, USA), anti-Myc (9E10, Santa Cruz) or anti-Flag M2 (Sigma) antibodies, then incubated for 1 h with Protein-A Sepharose, and washed three times using immunoprecipitation buffer without NP-40. For western blot, anti-TGFBI (ab155426, Abcam, Cambridge, UK: 1/1 000), anti-pan AKT (40D4, #2920, Cell Signaling Technology, Danvers, MA, USA: 1/1 000), anti-phospho AKT-S473 (#9271, Cell Signaling: 1/1 000), anti-PTEN (138G6, #9559, Cell Signaling: 1/1 000), anti-phospho PTEN S380 (#9551, Cell Signaling: 1/1 000), anti-TGFβ (#3711, Cell Signaling: 1/1 000), anti-Smad2 (D43B4, #5339, Cell Signaling: 1/1 000), anti-phospho Smad2-S465/467 (138D4, #3108, Cell Signaling: 1/1 000), anti-p38 MAPK (#9202, Cell Signaling; 1/1 000), anti-p44/42 MAPK (#9102, Cell Signaling, 1/1 000), anti-phospho p44/42-Thr202/Tyr204 (#9101, Cell Signaling, 1/1 000), anti-SAPK/JNK (56G8, #9258, Cell Signaling, 1/1 000), anti-phospho SAPK/JNK-Thr183/Tyr185 (#9251, Cell Signaling, 1/1 000), anti-Myc (Santa Cruz: 1/5 000), anti-Flag M2 (Sigma: 1/5 000), and anti-β-actin (Sigma: 1/5 000) antibodies were used. Please note that the phosphorylated form of the MAPK proteins required the use of the SuperSignal West Femto ECL (Pierce, #34095), whereas all other proteins were detected using normal Pierce ECL (#32209).

### IHC

Formalin-fixed and paraffin-embedded (FFPE) tumor sections were stained after microwave antigen retrieval using 0.01 M citric acid (pH 6.0) in combination with LSAB + System-HRP (Dako, Les Ulis, France). Rabbit polyclonal anti-TGFBI (HPA-017019, Sigma: 1/300) antibody was used. Primary antibody was omitted as a negative control. Tumor cytoplasmic staining was scored according to intensity of immunoexpression, with 0, 1, 2, and 3 indicating negative, weak, moderate, and strong staining of tumor cells, respectively. IHC scoring was performed blinded, without prior knowledge of clinicopathological parameters; each tissue section was scored twice and the result was then averaged.

### qRT-PCR

RNA was isolated using the Total RNA Purification Kit (Norgen Biotek, Thorold, Canada). One microgram of the total RNA was reverse-transcribed using the iScript cDNA Synthesis Kit (BioRad, Hercules, CA, USA). qRT-PCR was performed using either the SYBR Green PCR Master Mix (Life Technologies) or the SYBR Green (Roche) with the following primers: TGFBI (Forward: GTCCACAGCCATTGACCTTT, Reverse: GAGTTTCCAGGGTCTGTCCA); PTEN (Forward: TTGGCGGTGTCATAATGTCT, Reverse: GCAGAAAGACTTGAAGGCGTA); PAI-1 (Forward: GGCCATTACTACGACATCCTG, Reverse: GGTCATGTTGCCTTTCCAGT); ZEB1 (Forward: GTTCTGCCAACAGTTGGTTT, Reverse: GCTCAAGACTGTAGTTGATG); VIM (Forward: TGACCTCTCTGAGGCTGCCAACC, Reverse: TTCCATCTCACGCATCTGGCGCTC); CDH1 (Forward: CACCCTGGCTTTGACGCCGA, Reverse: AAAATTCACTCTGCCCAGGACGCG); CDH2 (Forward: CGCCATCCAGACCGACCCAA, Reverse: GTCGATTGGTTTGACCACGGTGAC); β-actin (Forward: AGAGCTACGAGCTGCCTGAC, Reverse: AGCACTGTGTTGGCGTACAG); GAPDH (Forward: TGTTGCCATCAATGACCCCTT, Reverse: CTCCACGACGTACTCAGCG); and HPRT (Forward: ATGAACCAGGTTATGACCTTGAT, Reverse: CCTGTTGACTGGTCATTACAATA). The 2^−ΔΔ^Ct method was used to calculate the relative miRNA expression^64^. The mRNA expression levels were normalized to the average expression of three housekeeping genes (β-actin, GAPDH, and HPRT).

### Proliferation, viability, caspase-3 activity, and clonogenic assays

For cell viability, the cells were plated at a density of 2000 cells per well in 96-well plates. Cell viability was evaluated using the ATPlite 1 Step Luminescence Assay System (PerkinElmer, Waltham, MA, USA). To assess cisplatin resistance, cells were exposed to varying concentrations of cisplatin (CDDP) for 72 h. Caspase-3 activity was assayed 20 h after cisplatin treatment and/or 48 h after transfection using the Caspase-3 Fluorometric Kit (BioVision, Milpitas, CA, USA).

For clonogenic assays, 500 cells per well were seeded in 6-well plates and incubated for 24 h, then treated with cisplatin at varying doses. After 10 days, colonies (>50 cells) were fixed with methanol and stained with crystal violet, then counted. It is important to note that C666-1 cells are not suitable for clonogenic assays as they do not form colonies; only NP69 cells were used for this assay.

### Luciferase reporter assay

MiR-449b/TGFBI binding activity: the 3′ untranslated region (3′UTR) of TGFBI containing the putative wild-type (WT) target site for miR-449b was cloned into the pMIR-REPORT vector (Ambion) (pMIR-TGFBI 3′UTR WT = CTAGTGCTCAGCGGATGTCACTGCCTGACATTCA). A mutant sequence was also generated (pMIR-TGFBI 3′UTR Mut = CTAGTGCTCAGCGGATGTGTGACGGAGACATTCA). HEK293T cells were reverse transfected with pre-miR-control and pre-miR-449b using JetPRIME; 24 h later, pMIR-TGFBI 3′UTR WT and pMIR-TGFBI 3′UTR Mut plasmids were co-transfected with pRL-SV Renilla vector (Promega, Madison, WI, USA), also using JetPRIME. After another 24 h, firefly and Renilla luciferase activities were measured using the Dual-Luciferase Reporter Assay (Promega).

TGFβ activity pathway: HEK293T was co-transfected with pSBE4-Luc vector or pSBE-Luc vector and pRL-SV Renilla vector, in addition to either pro-TGFβ1, TGFBI, ITGB3, ITGB5ΔC, or control plasmids, as indicated in the figure legends.

### Patient samples

Approval for the study was obtained from the Institutional Research Ethics Board (REB) at the Princess Margaret (PM) Cancer Center. Diagnostic FFPE blocks were collected for NPC patients treated at the PM between 1993 and 2009 and the RNA was extracted, as previously described^36^.

### RNA-seq and data analysis

For each NPC sample, 200 ng of input RNA was prepared using the Ribo-Zero Gold rRNA Removal Kit (Illumina, San Diego, CA, USA), followed by library preparation using the TruSeq Stranded Total RNA Sample Prep Kit (Illumina). Libraries were sequenced to >100 million paired-end 100 bp reads on the Illumina HiSeq 2000. The reads were then aligned using STAR^65^ (v2.4.2a), and the expression values were summarized using RSEM^66^ (v1.2.21).

### Statistical analyses

The statistical significance between groups was determined using either the ANOVA test, followed by the Bonferroni post-test when applicable or the Mann–Whitney *U*-test (socscistatistics.com). Data were represented as the mean ± SEM. All experiments were performed at least three times using new frozen batches of cells to maintain independence between replicates. Analysis and graphs were completed using GraphPad Prism software.

## Electronic supplementary material

Supplementary Figure Legends

Supplementary Figure 1

Supplementary Figure 2

Supplementary Figure 3

Supplementary Figure 4

Supplementary Figure 5
