# Supplementary material for: Dysregulation of the MiR-449b target TGFBI alters the TGFβ pathway to induce cisplatin resistance in nasopharyngeal carcinoma
Source: Oncogenesis. 2018 May 22;7(5):40. doi: 10.1038/s41389-018-0050-x (PMC5966388; doi:10.1038/s41389-018-0050-x)
Supplement: Supplementary file 1 — Supplementary Figure Legends [file 41389_2018_50_MOESM1_ESM.docx]

**SUPPLEMENTARY FIGURE LEGENDS**

**Figure S1**. NPC cell line C666-1 exhibits high miR-449b expression, high AKT expression, and cisplatin resistance compared to normal cell lines. (a) Relative expression of miR-449b assessed by qRT-PCR in NP69, C666-1 and NP460 cell lines, normalized to NP69 cells. (b) Cell viability was assessed using the ATPlite assay 72h after cisplatin treatment in NP69, C666-1, and NP460 cells. (c) Caspase-3 activity of NP69, C666-1, and NP460 cells 20h after cisplatin treatment. (d) Western blot was performed on the whole cell lysates (WCLs) of NP69, C666-1, and NP460 cells, using anti-ERK1/2 (αtotal-P44/42), anti-phospho ERK1/2 (T202/Y204; αpP44/42 (T202/Y204)), anti-phospho SAPK/JNK (T183/Y185; αpSAPK/JNK (T183/Y185)), anti-total SAPK/JNK (αtotal-SAPK/JNK), anti-p38 (αtotal-p38), with anti-β-actin (αβ-actin) as a loading control. (e) Western blot was performed on the whole cell lysates (WCLs) of NP69-miR-449b (or control). Immunoblots were performed with anti-ERK1/2 (αtotal-P44/42), anti-phospho ERK1/2 (T202/Y204; αpP44/42 (T202/Y204)), anti-phospho SAPK/JNK (T183/Y185; αpSAPK/JNK (T183/Y185)), anti-total SAPK/JNK (αtotal-SAPK/JNK), anti-p38 (αtotal-p38), with anti-β-actin (αβ-actin) as loading control. (f) Western blot was performed on the whole cell lysates (WCLs) of NP69, C666-1, and NP460 cells, using anti-PTEN (αtotal-PTEN), anti-phospho PTEN (S380; αpPTEN (S380)), anti-phospho AKT (S473; αpAKT (S473)), anti-pan AKT (αpan-AKT), with anti-β-actin (αβ-actin) as loading control. (g) Cell viability was assessed by ATPlite assay 72h after MK-2206 and/or cisplatin treatment in NP69-miR-449b (or control) stable cell lines. The data are expressed as the mean + SEM of at least three independent experiments. * P<0.05; ** P<0.01; *** P<0.001.

**Figure S2.** Regulation of TGFBI expression in NPC cells. (a) Table showing the putative genes regulated by miR-449b in NPC. (b) Relative expression of TGFBI assessed by qRT-PCR after transient transfection of pre-miR-449b (20 nM) and a non-targeting control (20 nM). The data are expressed as the mean + SEM of at least three independent experiments. *** P<0.001. (c) Western blot was performed on the supernatant of NP69-miR-449b stable cells incubated in MEM for 48h; Ponceau S was used to demonstrate equal amounts of protein (10 µg) for each condition. (d) Relative expression of TGFBI assessed by qRT-PCR on stable NP69-anti-miR-449b cells (and control) (top), and after transient transfection of miR-449b inhibitor (100 nM) and a non-targeting control (100 nM) (bottom). The data are expressed as the mean + SEM of at least three independent experiments. * P<0.05; *** P<0.001. (e) Western blot was performed on the whole cell lysates (WCLs) (left) and supernatant (right) of NP69-anti-miR-449b (or control), after 48h of incubation in MEM. Immunoblots were performed with anti-TGFBI (αTGFBI), and with anti-β-actin (αβ-actin) as loading control (left). Ponceau S was used to demonstrate equal amounts of protein (10 µg) for each condition (right). (f) Cell viability was assessed by ATPlite assay 72h after cisplatin treatment in NP69-antimiR-449b (or control) stable cell lines. (g, and h) All experiments were performed on NP69-shTGFBI (-1, -2) stable cell lines generated by lentivirus infection and incubated in MEM for 48h. (g) Relative expression of TGFBI assessed by qRT-PCR, normalized to shRNA control cells. The data are expressed as the mean + SEM of at least three independent experiments. *** P<0.001. (h) Western blot was performed on cell supernatant using anti-TGFBI (αTGFBI) antibody; Ponceau S was used to demonstrate that equal amounts of protein (10 µg) were loaded for each condition. Differences observed in the Ponceau stain were likely due to the high amount of 70 kDa proteins secreted by C666-1 cells.

**Figure S3.** (a) Relative expression of TGFBI assessed by qRT-PCR in NP69, C666-1, and NP460 cell lines, normalized to NP69. (b) Western blot was performed on the supernatant of NP69, C666-1, and NP460 cells using anti-TGFBI (αTGFBI) antibody; Ponceau S was used to show equal amounts of protein loaded in each condition. (c) IHC was performed on NPC patient samples using anti-TGBI polyclonal antibody (Sigma). TGFBI was observed in both the cytoplasm and the plasma membrane of tumor cells, as well as in stromal cells. Tumour cell staining was quantified as described. This figure demonstrates representative photomicrographs of TGFBI-expressing tumour of score 1, 2, and 3. (d) Original photographs representing the morphology of NP69-miR-control *vs.* NP69-miR-449b stable cell lines. (e and f) Relative expression of ZEB1, vimentin (VIM), E-cadherin (CDH1), and N-cadherin (CDH2) as assessed by qRT-PCR on NP69-miR-449b (e) or NP69-shTGFBI (f) stable cells incubated in MEM for 48h. The data are expressed as the mean + SEM of at least three independent experiments. * P<0.05; ** P<0.01; *** P<0.001. (g) Western blot of NP69 and C666-1 cells; C666-1 cells were transfected with empty vector (CTL) or TGFBI-encoding vector (Myc-tagged; TGFBI-Myc). Media was replaced by MEM 6h after transfection. WCLs were collected after 48h of incubation. (h) NP69-miR-449b stable cells were treated with MK-2206, an AKT inhibitor, at 100 nM, 1 µM, or 4 µM for 72h. Immunoblots were performed with anti-phospho AKT (S473; αpAKT (S473)), and anti-pan AKT (αpan-AKT) to assess the efficiency of the inhibitor, while anti-ZEB1 (αZEB1), anti-CDH1 (αCDH1), and anti-CDH2 (αCDH2) antibodies were used as indicators of EMT. Anti-β-actin (αβ-actin) antibody was used as loading control. The data are expressed as the mean + SEM of at least three independent experiments.

**Figure S4.** (a) Co-immunoprecipitations were performed on HEK293T cells expressing ITGB3 alone or with TGFBI in the absence (TGFBI) or presence of pro-TGFβ1 (TGFBI+ TGFβ1-Flag). These data show that pro-TGFβ1 is not able to displace TGFBI-ITGB3 binding. (b) Co-immunoprecipitations were performed on HEK293T expressing ITGB5ΔC alone or with TGFBI in the absence (TGFBI-Myc) or presence of pro-TGFβ1 (TGFBI-Myc+ TGFβ1-Flag). These data show that pro-TGFβ1 is not able to displace TGFBI-ITGB5 binding. For a and b, Western blot of the whole cell lysates (WCLs) before pull-down is shown as a control for specificity and expression. (c) Relative expression of PAI-1 assessed by qRT-PCR in HEK293T cells transiently transfected with pro-TGFβ1-encoding plasmid (1 µg) and TGFBI-Myc encoding plasmid (0.5-2 µg) as indicated (normalized to control cells; CTL). (d) Relative luciferase activity was assessed after transient transfection of pro-TGFβ1-Flag (1 µg) and the co-transfection of pSBE4-luciferase vector (150 ng) and Renilla plasmid (100 ng) with or without SB431542 treatment (5 µM) (left). Relative luciferase expression assessed after transient transfection of pro-TGFβ1 and co-transfection of pSBE-luciferase vector (150 ng) and Renilla plasmid (50 ng) (right). (e) Relative luciferase activity was assessed after transient transfection of ITGB3 (1 µg) or ITGB5 (1 µg) and the co-transfection of pSBE4-luciferase vector (150 ng) and Renilla plasmid (100 ng) with or without SB431542 treatment (5 µM). Note that, as these experiments were conducted as the same time as those for Figure 5h, the values for ITGB3 and ITGB5 condition alone are the same for both figures (left). Relative luciferase expression was assessed after transient transfection of ITGB3 (1 µg) or ITGB5ΔC (1 µg) and co-transfection of pSBE-luciferase vector (150 ng) and Renilla plasmid (50 ng) (right). The data are expressed as the mean + SEM of at least three independent experiments. ** P<0.01; *** P<0.001.

**Figure S5.** Western blot quantification. The relative expression of the proteins of interest were assessed using Image J. For phosphorylated proteins, their corresponding non-phosphorylated form and β-actin were used as controls (formula: phosphorylated protein/total protein/β-actin). For all other proteins, only β-actin was used as control. The data are expressed as the mean + SEM of at least three independent experiments. * P<0.05; ** P<0.01; *** P<0.001.
