# Supplementary figures and images for: Dysregulation of the MiR-449b target TGFBI alters the TGFβ pathway to induce cisplatin resistance in nasopharyngeal carcinoma
Source: Oncogenesis. 2018 May 22;7(5):40. doi: 10.1038/s41389-018-0050-x (PMC5966388; doi:10.1038/s41389-018-0050-x)

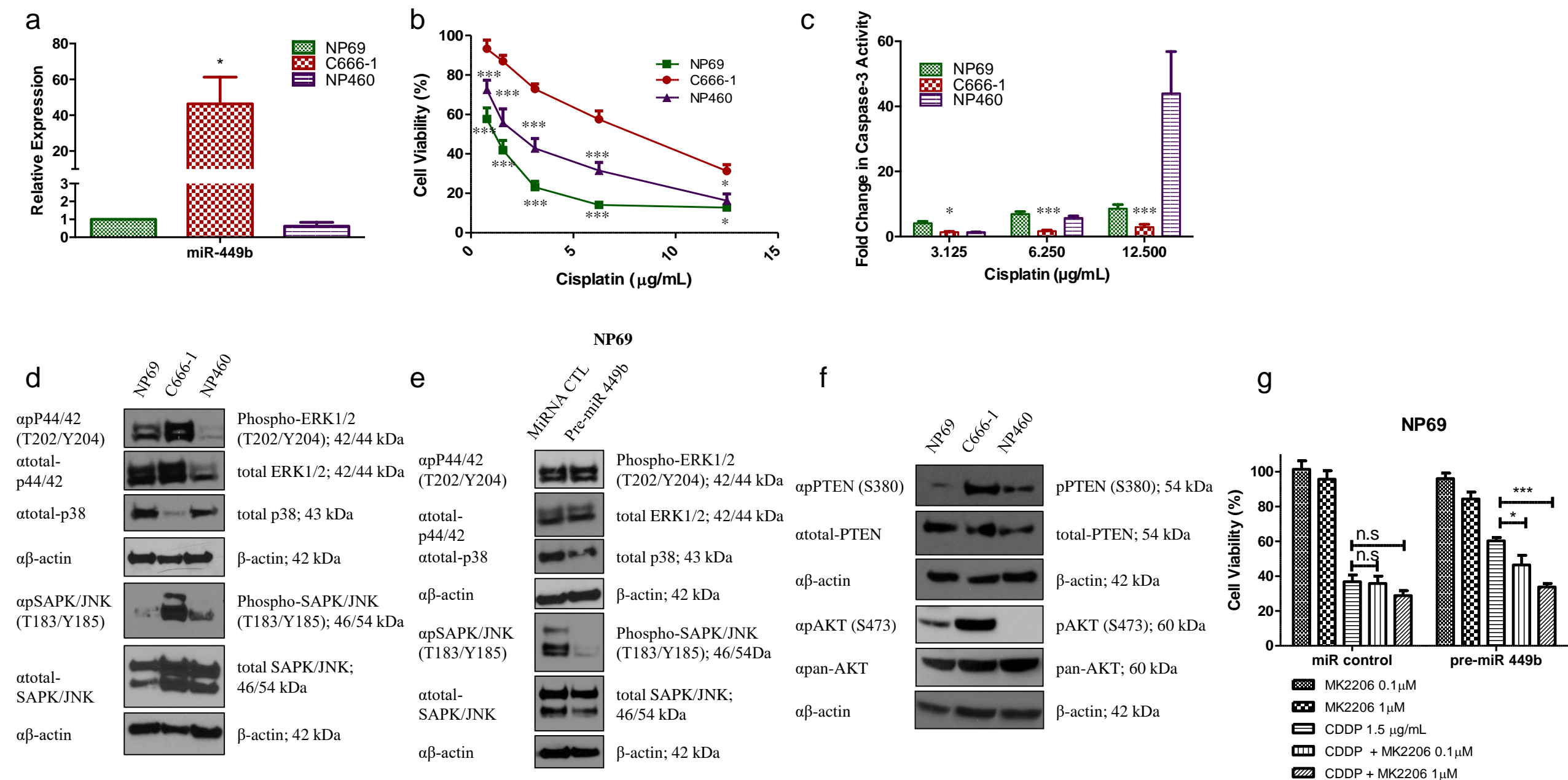

Figure S1.

Supplement: Supplementary file 2 — Supplementary Figure 1 [file 41389_2018_50_MOESM2_ESM.pdf]

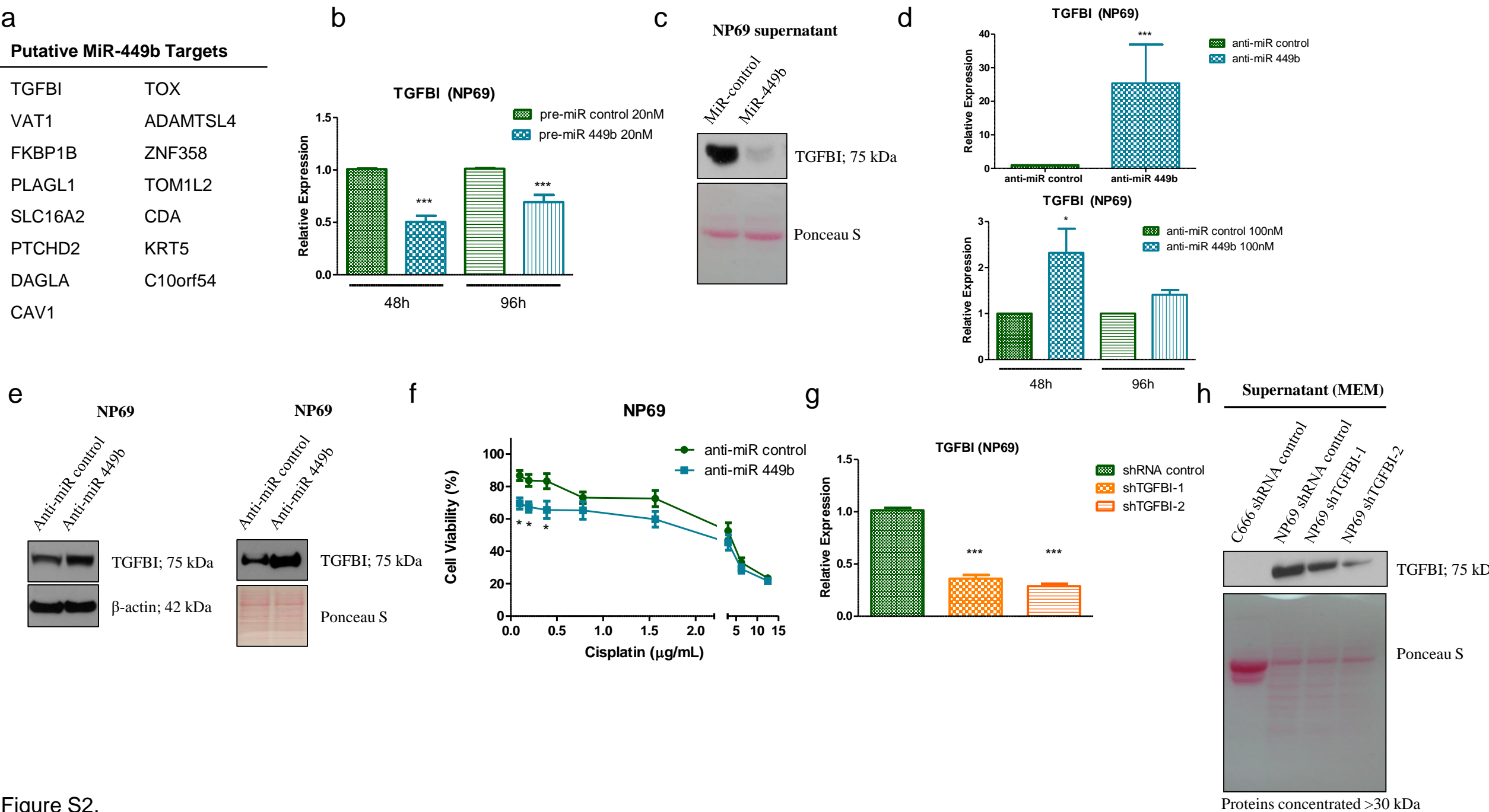

Figure S2.

Supplement: Supplementary file 3 — Supplementary Figure 2 [file 41389_2018_50_MOESM3_ESM.pdf]

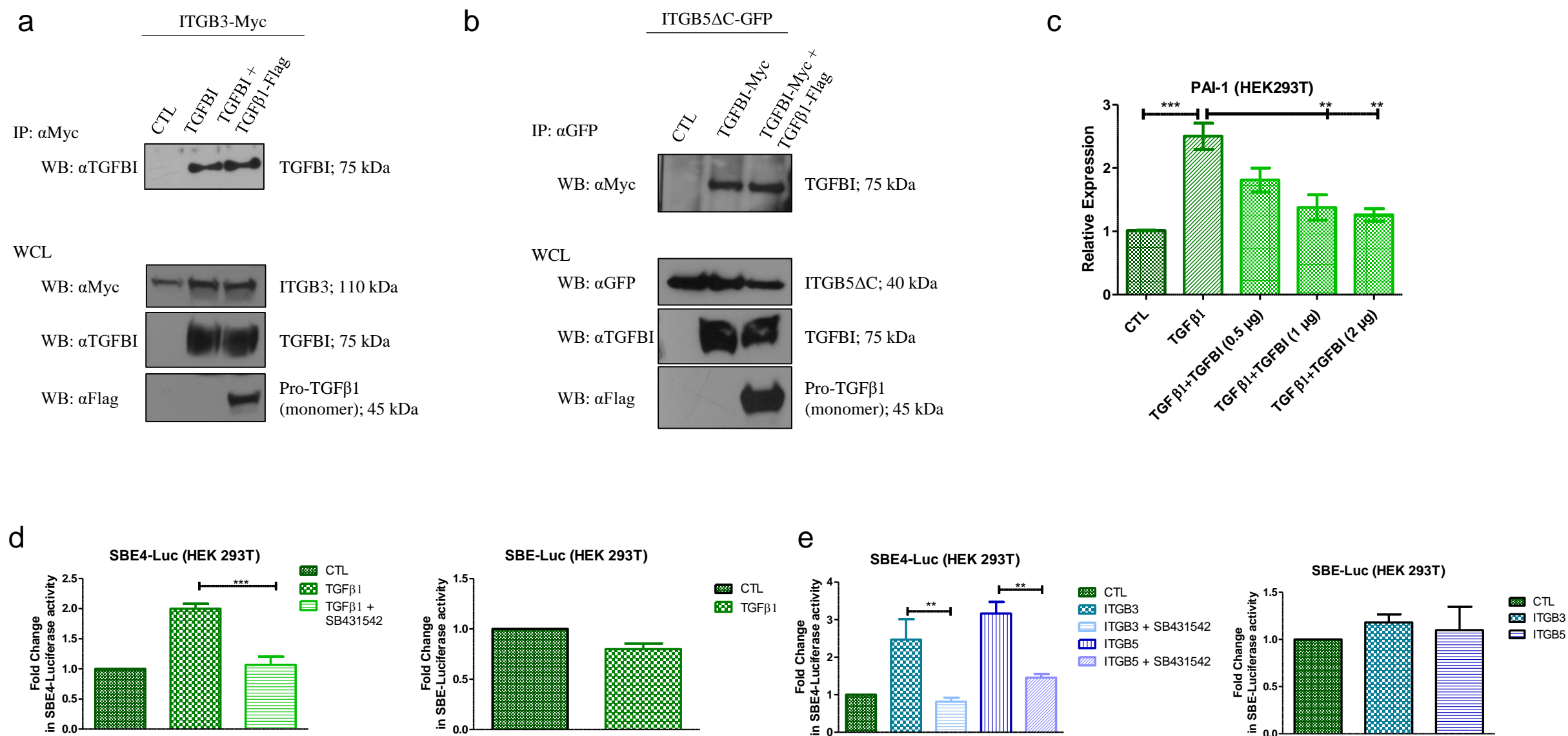

Figure S4.

Supplement: Supplementary file 5 — Supplementary Figure 4 [file 41389_2018_50_MOESM5_ESM.pdf]

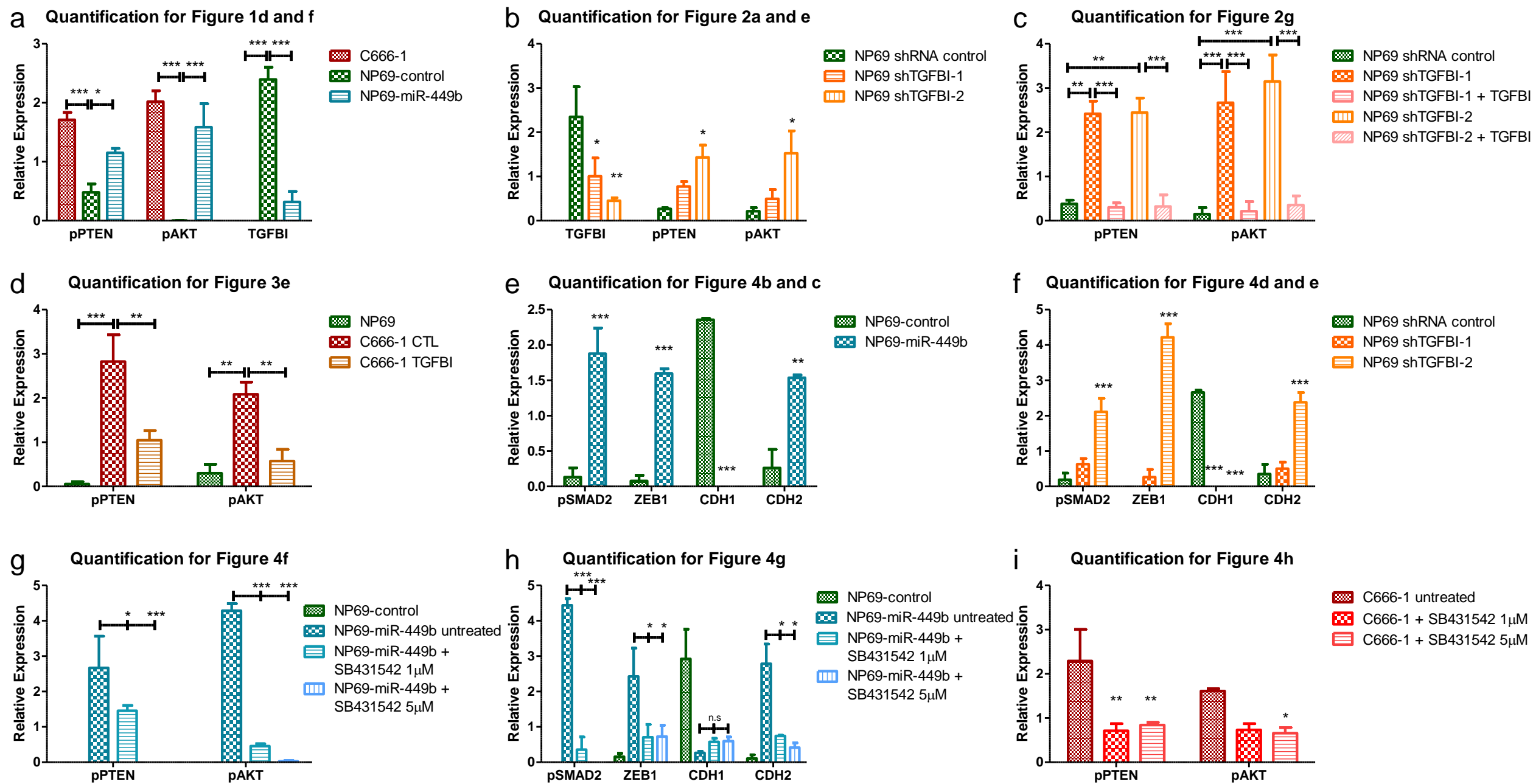

Figure S5.

Supplement: Supplementary file 6 — Supplementary Figure 5 [file 41389_2018_50_MOESM6_ESM.pdf]
