# Supplementary material for: Dysregulation of the MiR-449b target TGFBI alters the TGFβ pathway to induce cisplatin resistance in nasopharyngeal carcinoma
Source: Oncogenesis. 2018 May 22;7(5):40. doi: 10.1038/s41389-018-0050-x (PMC5966388; doi:10.1038/s41389-018-0050-x)
Supplement: Supplementary file 4 — Supplementary Figure 3 [file 41389_2018_50_MOESM4_ESM.pdf]

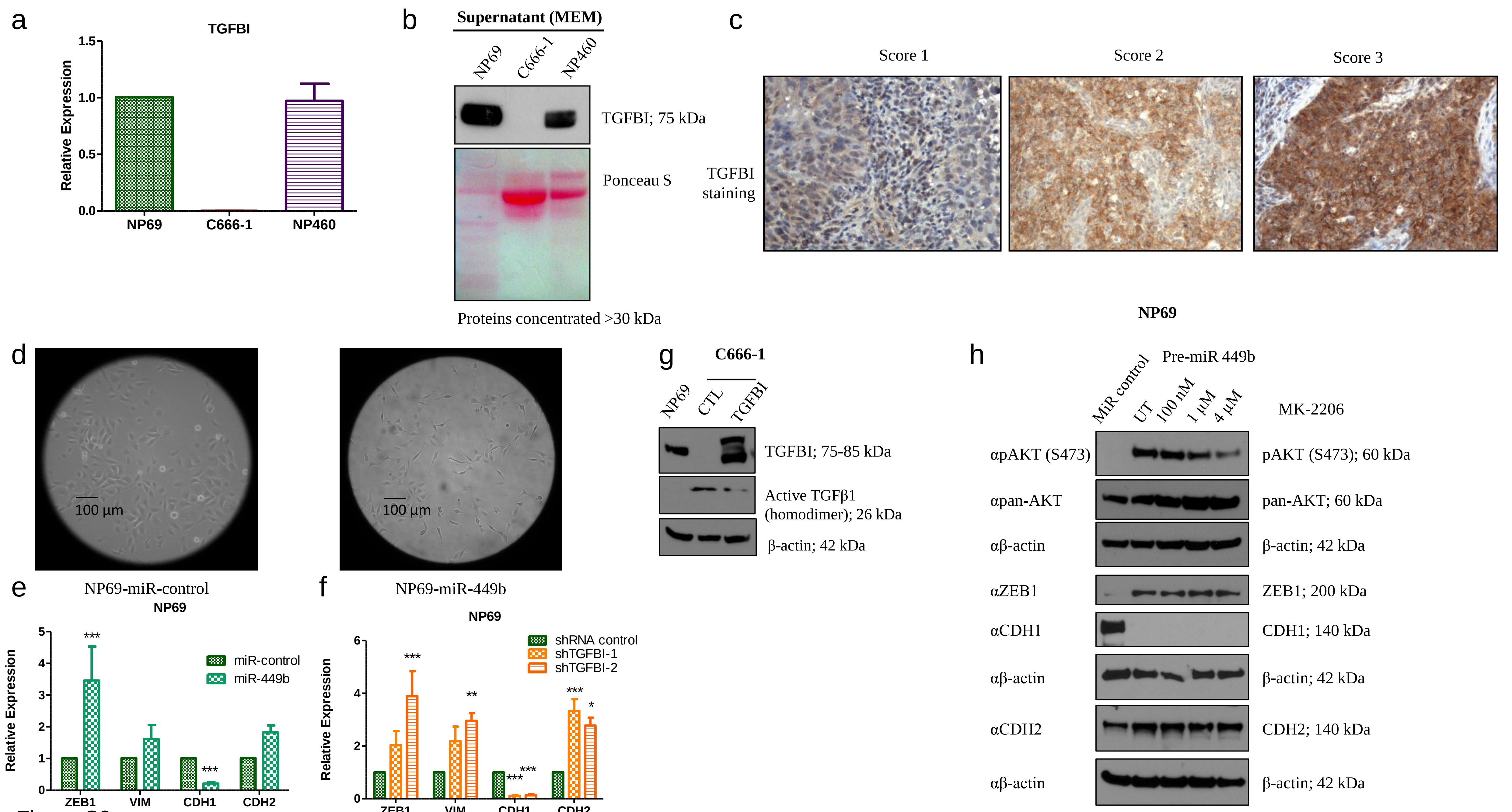

a
TGFBI
Relative Expression
1.5
1.0
0.5
0.0
NP69
C666-1
NP460
b
Supernatant (MEM)
NP69
C666-1
NP460
TGFBI; 75 kDa
Ponceau S
Proteins concentrated >30 kDa
c
Score 1
Score 2
Score 3
TGFBI staining
d
100 µm
100 µm
NP69-miR-control
NP69-miR-449b
e
NP69
miR-control
miR-449b
Relative Expression
ZEB1
VIM
CDH1
CDH2
f
NP69
shRNA control
shTGFBI-1
shTGFBI-2
Relative Expression
ZEB1
VIM
CDH1
CDH2
g
C666-1
NP69
CTL
TGFBI
TGFBI; 75-85 kDa
Active TGFβ1 (homodimer); 26 kDa
β-actin; 42 kDa
h
NP69
Pre-miR 449b
MiR control
UT
100 nM
1 µM
4 µM
MK-2206
αpAKT (S473)
pAKT (S473); 60 kDa
αpan-AKT
pan-AKT; 60 kDa
αβ-actin
β-actin; 42 kDa
αZEB1
ZEB1; 200 kDa
αCDH1
CDH1; 140 kDa
αβ-actin
β-actin; 42 kDa
αCDH2
CDH2; 140 kDa
αβ-actin
β-actin; 42 kDa

Figure S3.
